# A Simple Method for In-Depth Proteome Analysis of Mammalian Cell Culture Conditioned Media Containing Fetal Bovine Serum

**DOI:** 10.3390/ijms22052565

**Published:** 2021-03-04

**Authors:** Ren Nakamura, Daisuke Nakajima, Hironori Sato, Yusuke Endo, Osamu Ohara, Yusuke Kawashima

**Affiliations:** 1 Department of Applied Genomics, Kazusa DNA Research Institute, Kisarazu, Chiba 292-0818, Japan; r-nakamu@kazusa.or.jp (R.N.); nakajima@kazusa.or.jp (D.N.); hsato@kazusa.or.jp (H.S.); ohara@kazusa.or.jp (O.O.); 2 Department of Frontier Research and Development, Kazusa DNA Research Institute, Kisarazu, Chiba 292-0818, Japan; endo@kazusa.or.jp

**Keywords:** proteome analysis, secreted proteins, albumin removal, conditioned medium, fetal bovine serum, DIA

## Abstract

A conditioned medium of a cell culture is widely used for various biological applications and frequently analyzed to characterize the functional proteins responsible for observed biological functions. However, a large number of abundant proteins in fetal bovine serum (FBS), usually included in the conditioned medium of a mammalian cell culture medium, hampers in-depth proteomic analysis by liquid chromatography–tandem mass spectrometry (LC-MS/MS). For a deep proteomic analysis of a conditioned medium by LC-MS/MS, we developed a simple albumin depletion approach coupled with data-independent acquisition (DIA)-mode LC-MS/MS for the conditioned medium of mammalian cells in this study. The results showed that this approach enabled the detection of more than 3700 cell-derived proteins in the cell culture supernatant containing FBS. We further demonstrated the potency of this approach by analyzing proteins in the conditioned media of HeLa cells with and without tumor necrosis factor (TNF) stimulation: >40 differentially accumulated proteins, including four cytokines, upon TNF stimulation were identified in the culture media, which were hardly detected by conventional proteome approaches in the literature.

## 1. Introduction

Conditioned media are widely used in cell biology because they frequently exert characteristic functions depending on their preparation conditions. In this regard, the conditioned media also serve as valuable sources to seek the functional entities responsible for the observed biological function of interest by proteome analysis of the conditioned media using liquid chromatography–mass spectrometry (LC-MS/MS). However, this approach is not always successful because the conditioned media contain a high concentration of fetal bovine serum (FBS), which hampers the detection of proteins at low concentrations by LC-MS/MS. To solve this problem, various attempts have been made in the literature [1,2,3,4,5,6]. Nevertheless, the simplest approach is the depletion of abundant proteins in FBS, which is still the method of choice for proteome analysis of the conditioned media in plasma/serum analysis.

The depletion of albumin, the most abundant protein in FBS, is conventionally conducted using antibodies. According to this approach, the proteins of interest are obtained by negative selection with an antibody specific to albumin. However, the antibody columns for the bovine serum/plasma are commercially unavailable. The other method, which takes advantage of the positive selection of the proteins of interest, is commercially available (ProMax Albumin Removal kit, Polysciences, Warrington, PA, USA). This approach is simple and probably applicable to depleting the albumin derived from several mammals. However, to our best knowledge, there is no report about this albumin depletion method’s performance on LC-MS/MS-based proteome analysis. Here, we thus examined this albumin depletion approach in terms of LC-MS/MS-based proteome analysis performance. We then devised a simple method that enabled us to conduct an in-depth proteome analysis of the conditioned media of cultured mammalian cells by combining the albumin depletion kit with data-independent acquisition (DIA)-mode LC-MS/MS. This method’s potency was demonstrated by the detection of the TNF-induced proteins of the HeLa cells cultured in the presence of FBS.

## 2. Results and Discussion

### 2.1. Evaluation of FBS-Derived Albumin Depletion for Proteome Analysis

**Figure 1:**
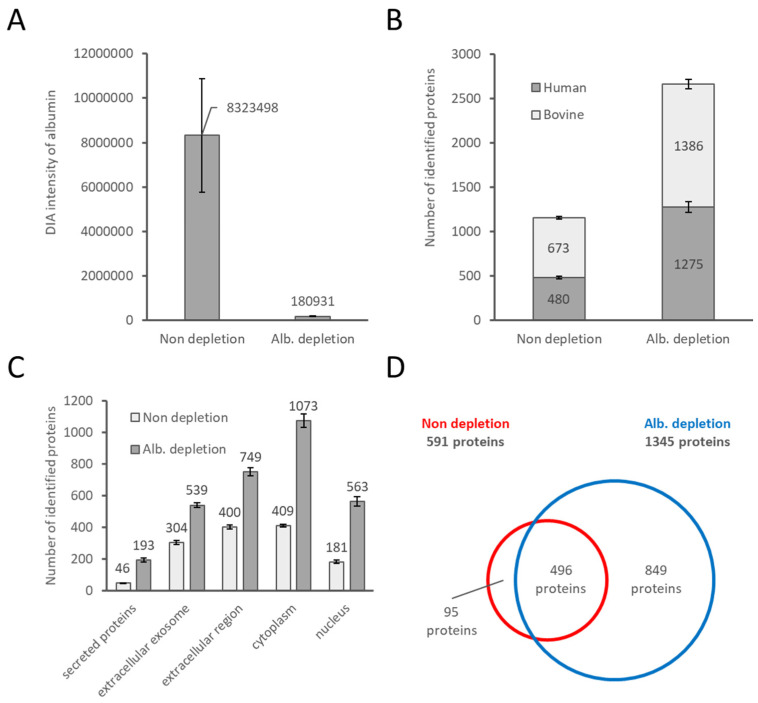
Effect of albumin depletion in the FBS-containing culture supernatant for proteome analysis. The undepleted and albumin-depleted samples were analyzed, with *n* = 3 each. (**A**) Amount of albumin by non-depletion and albumin depletion when normalized by the equivalent volume of the supernatant was analyzed by LC-MS. (**B**) Bar graph of the number of human and bovine proteins observed by proteome analyses. (**C**) Comparison of cellular localization by gene ontology (GO) enrichment analysis for the identified human proteins. (**D**) Venn diagram of the number of human proteins observed by proteome analyses. For (**B**,**C**), the results of the MS data individually analyzed by the Scaffold DIA software were used. For (**D**), the results of the simultaneously analyzed MS data collected for each treatment by the Scaffold DIA were used. Abbreviations: ALB, serum albumin.

To evaluate the effect of bovine albumin depletion by the ProMax Albumin Removal kit for proteome analysis, we compared the DIA-mode LC-MS/MS data of the HeLa cell culture supernatants cultured in 10% FBS-containing medium with and without the ProMax treatment. As shown in Figure 1A, approximately 98% of the bovine albumin was removed by ProMax, indicating a highly efficient bovine albumin removal using this kit, as shown by the supplier. We next examined the number of proteins identified by DIA-mode LC-MS/MS. It was revealed that, on average, 2.6-fold more human proteins derived from HeLa cells were detected in the albumin-depleted samples than in the undepleted ones. This enhanced detection sensitivity is not confined to human proteins; the number of FBS-derived bovine proteins was twice higher in the albumin-depleted supernatant than in the depleted ones, on average (Figure 1B). The number of both human- and bovine-derived proteins increased because the dynamic range of protein concentration in the supernatant could be considerably narrowed by depleting FBS-derived albumin, which is the most abundant protein in the culture supernatant. Figure 1C shows the cellular localization of the human proteins identified by each treatment.

The conditioned medium is expected to contain all the proteins released from the cultured cells, implying that some intracellular proteins released by necrotic and/or apoptotic cells also could be found in the conditioned medium. This is actually the case because we found the presence of approximately 1% of dead cells (measured by a trypan blue staining assay; about 2–3% of apoptotic cells measured by fluorescence flow cytometry) under the culture conditions of the HeLa cells in this study (Supplementary Figure S1). Thus, we consider that the considerable number of cytoplasmic and nuclear proteins in the conditioned medium in our study as well as previous reports [7,8,9] might be released from dead cells although we did not experimentally confirm the origin of these proteins in this study. It is interesting to note that proteins with signal peptide, which are regarded as authentic secretory proteins through the classical secretory pathway, occupied only 42.3% and 32.9% of the total proteins detected in the conditioned medium before and after the albumin depletion in this study, respectively (Figure 1C and Supplementary Table S1). We consider that the lower occurrence rate of the authentic secretory proteins in the detected protein list after the albumin depletion probably resulted from the increase in the number of cytoplasmic and nuclear proteins due to the improved protein detection sensitivity by decreasing the dynamic range of protein concentrations in the sample.

Figure 1D and Supplementary Table S1 show a Venn diagram of the protein ensembles detected before and after albumin depletion. Approximately 84% of the proteins identified before albumin depletion were certainly detected after albumin depletion, although the remaining proteins (95 proteins) were missing after the albumin depletion. The results indicated the albumin depletion by positive selection using the ProMax kit resulted in loss of a small but considerable number of proteins in the conditioned medium. Supplementary Table S2 shows a list of proteins, which seem to be lost by albumin depletion. Although we cannot identify any common characteristics in these proteins, we consider three reasons to explain the loss of these proteins: (1) incapability to bind to the ProMax beads; (2) incapability of elution from the ProMax beads; and (3) a strong association with albumin. Furthermore, 77% of the lost proteins were extracellularly localized, leading to the loss of interesting proteins. Although the depletion method is superior considering the number of observed proteins, by implementing the depletion and the non-depletion methods, the observed proteins can be complemented. It is important to keep the loss of these proteins during albumin depletion in mind. Still, the depletion of albumin by ProMax provided us with great depth enhancement of the proteome analysis by LC-MS/MS. Therefore, we consider that the albumin depletion by ProMAX could be a powerful approach for proteome analysis of culture supernatants containing FBS, as an alternative to the antibody-assisted one.

### 2.2. Further Depth Enhancement of Proteome Analysis by Changing the Volume of the Culture Media

**Figure 2:**
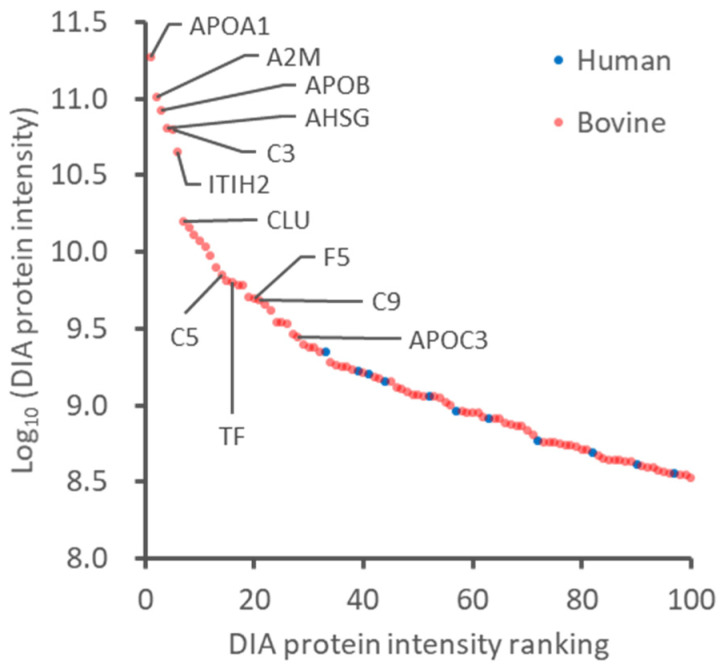
Protein concentration dynamic range of abundant proteins in the albumin-depleted supernatant. The top 100 proteins of average DIA protein intensity in the albumin-depleted samples were plotted. Red and blue dots represent bovine and human proteins, respectively.

The results described above indicated that albumin depletion improves the depth of proteome analysis by LC-MS/MS, probably because of the reduction of suppression effects of an extraordinarily abundant protein in the conditioned media. However, even excluding the FBS-derived albumin, the supernatant’s high abundance protein was still accounted for by FBS-derived bovine protein (Figure 2). There is no way to remove all of these high-abundance bovine proteins. It is difficult to narrow the dynamic range of protein concentrations in the supernatant by a simple pretreatment. In this study, as an alternative approach, we attempted to enrich secreted proteins by reducing the volume of culture medium and albumin depletion. HeLa cells at 70% confluency (approximately 6 × 10^6^ cells per dish) were cultured in 10, 5, and 2 mL of medium containing 10% FBS for 24 h, and then the supernatant was collected. In the 95-mm dish used, 10 mL of the medium is typically used. However, the entire surface of the dish was filled with 2 mL of the medium, and there were no changes in cell morphology and phenol red after one day. The number of dead and apoptotic cells was also counted in culture under the three conditions, confirming no significant change (Supplementary Figure S1).

**Figure 3:**
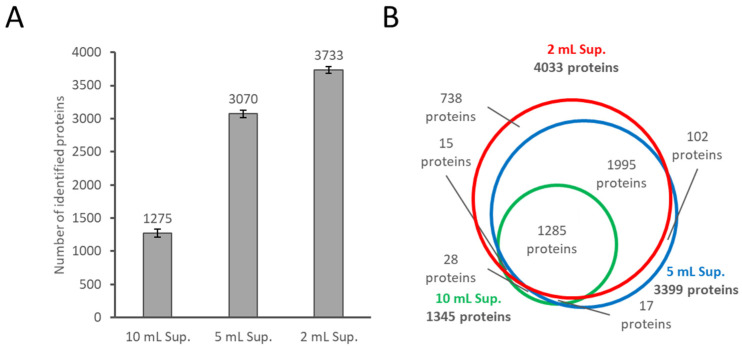
Effect of the volume of the medium used on proteome analysis. (**A**) Bar graph of the number of human proteins observed in albumin-depleted supernatant of cultured in 10, 5, and 2 mL of medium containing 10% FBS. (**B**) Venn diagram of the number of human proteins observed in the three treated samples. For (**A**), the results of the MS data individually analyzed by the Scaffold DIA software were used. For (**B**), the results of the simultaneously analyzed MS data collected for each treatment by the Scaffold DIA were used. Abbreviations: ALB, serum albumin; Sup, supernatant.

**Figure 4:**
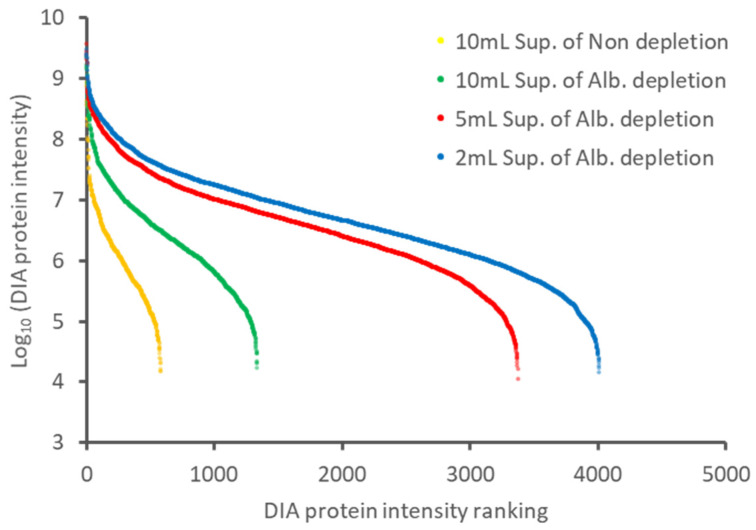
The dynamic range of protein concentration in the supernatant samples treated with different approaches. The proteins of an average DIA protein intensity in each treated sample were plotted. Yellow dots represent non-depletion of the supernatant cultured in 10 mL of the medium. Green, red, and blue dots represent albumin depletion of the supernatant cultured in 10, 5, and 2 mL of the medium, respectively. Abbreviations: ALB, serum albumin; Sup, supernatant.

The supernatants of each culture condition were subjected to albumin depletion followed by LC-MS/MS analysis. As shown in Figure 3A, the number of identified human proteins increased by reducing the volume of the culture media; the number of proteins detected was 2.4-fold in the 5 mL medium and 2.9-fold in the 2 mL medium, on average, compared to the 10 mL medium. The results indicated that the cell-derived proteins could be easily enriched by decreasing the volume of the medium. Figure 3B shows the overlap of the human proteins identified under the three conditions. The list of identified proteins is shown in Supplementary Table S3. The degree of overlap of the identified proteins in each condition is high, indicating that the protein profiling was not significantly affected by reducing the medium volume. Figure 4 shows the dynamic range of the identified proteins by DIA protein intensity. Since the same MS measurement method in each treatment was used, the dynamic range of the analysis naturally was almost unchanged. However, the detected proteins by the depletion of albumin and the culture medium’s reduction greatly increased. These results suggest that the depletion of albumin and the reduction of the culture medium reduced the dynamic range of the protein concentration in the sample as intended. Although care must be taken with the culture period, the reducing the volume of the medium is suitable for proteome analysis because it does not change the cell state substantially. We believe that the approach described above is an effective combination for in-depth proteome analysis of conditioned media. This approach allowed us to detect far more proteins (3700 proteins) than those reported by the results using protein fractionation (585 proteins) [6].

### 2.3. Reproducibility and Demonstration of the Potency of the Proteome Analysis, Including Albumin Depletion under Cell Culture Conditions with a Small Culture Media

In practical use, high quantitative reproducibility of the overall steps, albumin depletion, and LC-MS measurements is highly critical. The reproducibility of our proteome analysis was thus evaluated by calculating the coefficient of variation (CV) of the individual protein quantification values identified by each treatment (Supplementary Figure S2). The three treatments that adapted the albumin depletion had a median CV of less than 10%, and the supernatant cultured in 2 mL had the highest reproducibility with a median CV of 6.3%. The undepleted samples with the fewest work steps had a median CV of 18%, clearly higher than the others, and less reproducible. In the undepleted case, due to the wide dynamic range of concentration in the supernatant, many peptides were detected around the limit detection sensitivity of MS, resulting in poor reproducibility of the protein quantification values. Alternatively, in the albumin-depleted samples, the dynamic range of the protein concentration in the supernatant was reduced, and the number of peptides identified increased where there was a margin in the detection sensitivity of the MS. As a result, the overall protein quantification values were highly reproducible even with an increased albumin depletion step. The lowest CV of 2 mL was also due to the reduction of the dynamic range in the supernatant. The lowest median CV of the supernatant cultured in 2 mL was also due to the decrease in the supernatant’s dynamic range. From the above results, the reduction of the dynamic range of the protein concentration in the sample improved the detection of the cell-derived proteins and the reproducibility of proteome analysis. Therefore, our approach is an accurate technique for comparative quantitative analysis.

**Figure 5:**
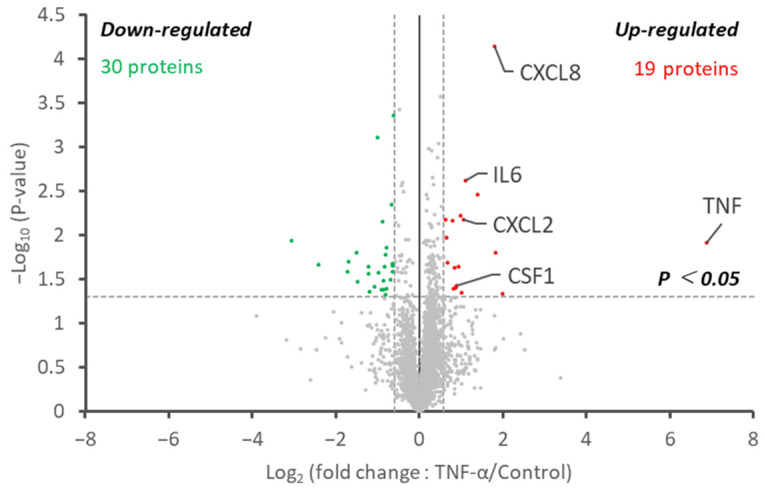
In-depth proteome analysis of TNF-stimulated and non-stimulated HeLa cells. The volcano plot represents the change in protein abundance upon TNF stimulation. Proteins with decreased and increased abundances are illustrated as green and red dots, respectively.

To evaluate the performance of our method developed in this study, we analyzed and compared the supernatant of tumor necrosis factor (TNF)-stimulated HeLa cells and non-stimulated HeLa cells. It is known that NF-κB is activated by TNF stimulation to induce the secretion of cytokines and chemokines, such as TNF, IL1, IL6, CXCL1, CXCL2, CXCL8, and CSF1 [10,11,12]. Figure 5 shows a volcano plot of the quantitative proteome data representing changes in a human-derived protein’s profile upon TNF stimulation. The list of identified proteins is shown in Supplementary Table S4. Unsurprisingly, a large difference in the amount of TNF was detected in the group to which TNF was added. In this experiment, it was impossible to distinguish between the added and secreted TNF, so it was unknown whether there was a change in the amount of secreted TNF. Besides this, 17 upregulated proteins and 27 downregulated proteins were significantly altered by TNF stimulation. The upregulated proteins included cytokines, such as CSF1, CXCL2, IL6, and CXCL8, and terms related to cytokine and chemokine activity were statistically significant in the GO enrichment analysis (Supplementary Figure S3). For the downregulated proteins, distinct categories could not be found. We also analyzed the supernatant of the TNF-stimulated HeLa and non-stimulated HeLa cells by the non-depletion method (Supplementary Table S5). No differences in CSF1 and CXCL2 were observed between the two groups in the non-depletion method, and no new altered cytokines and chemokines were found. The superiority of the depletion method was confirmed. Therefore, our proteome analysis has high proteome coverage even from the FBS-containing supernatant, and high-precision comparative analysis is possible, so we believe that it will be a powerful strategy for analyzing the supernatant proteins.

## 3. Materials and Methods

### 3.1. Cell Culture and Preparation of Conditioned Medium

HeLa cells were plated in 95-mm dishes (Nunc EasYDish; Thermo Fisher Scientific, Waltham, MA, USA) in serum media composed of minimum essential media (MEM; Thermo Fisher Scientific) containing 10% FBS (Thermo Fisher Scientific). Cell cultures were maintained in a CO_2_ incubator (astec, Fukuoka, Japan) maintained at 37 °C and in a humidified atmosphere of 95% air and 5% CO_2_. When the HeLa cells reached 70% confluence and the media exchange, three different conditioned media were adjusted. Other fluid volumes (10, 5, and 2 mL) of MEM containing 10% FBS were added to each dish and incubated. Twenty-four hours later, the total amount of cell culture supernatant was collected in each 15-mL tube (Eppendorf, Hamburg, Germany), and the cells were removed by centrifugation at 1000× *g* for 2 min.

The number of dead cells was counted using trypan blue (Sigma-Aldrich, St. Louis, MO, USA) according to the manufacturer’s instructions. In addition, apoptotic cells were detected using an Annexin V-fluorescein isothiocyanate (FITC) apoptosis detection kit II (BD Biosciences, Franklin Lakes, NJ, USA) according to the manufacturer’s instructions. Briefly, cells were washed with cold phosphate buffered saline (PBS; nacalai tesque, Kyoto, Japan) twice, and then cells were treated with propidium iodide (PI) and Annexin V-FITC in a binding buffer for 15 min. Prepared cells were analyzed using a FACSCelesta Flow Cytometer (BD Biosciences).

For the TNF stimulation test, the cell culture was divided into two groups of three dishes each: the control and TNF-stimulated groups. During media exchange, 2 mL of MEM containing 10% FBS was added to each dish. Human TNF (FUJIFILM Wako Pure Chemical Corp., Osaka, Japan) was added to the TNF-stimulation group at a final concentration of 10 ng/mL (media volume: 2 mL) and incubated for 24 h.

### 3.2. Preparation of Cell Culture Supernatants

The cell culture supernatant was prepared in two ways. In the first set (the albumin depletion method), 100 µL of cell culture supernatant was added to 350 µL of ProMax Binding/Wash Buffer and 20 µL of ProMax particles, followed by gentle mixing for 20 min at room temperature. The mixture sample collected the particles by magnetic separation and the supernatant containing albumin was removed. The particles were washed three times with 500 µL ProMax Binding/Wash Buffer. The particles were then mixed for 10 min at room temperature in 100 µL of 100 mM tris (hydroxymethyl) aminomethane (Tris)- hydrochloric acid (HCl) (pH 8.5) (NIPPON GENE, Tokyo, Japan) and 2% sodium dodecyl sulfate (SDS) (Promega, Madison, WI, USA). The supernatant was transferred to a fresh 1.5 mL tube by magnetically separating the particles. Although the manufacturer’s recommendations suggest optimization may be required depending on the application, using the protocol described here we found no further optimization was required for a wide range of samples. In the second set (non-depletion), 5 μL of cell culture supernatant in a 1.5 mL tube was diluted with 95 μL of 100 mM Tris-HCl (pH 8.5) and 2% SDS.

### 3.3. Protein Digestion

The pretreated cell culture supernatant was treated with 10 mM dithiothreitol (FUJIFILM Wako Pure Chemical Corp.) at 50 °C for 30 min and alkylated with 30 mM iodoacetamide (FUJIFILM Wako Pure Chemical Corp.)in the dark at room temperature for 30 min and subjected to clean up and digestion with the single-pot solid phase-enhanced sample preparation (SP3) [13]. Briefly, two types of beads (hydrophilic and hydrophobic Sera-Mag Speed-Beads; Cytiva, Marlborough, MA, USA) were used. These beads were combined at a 1:1 (*v*/*v*) ratio, rinsed with distilled water (DW; Thermo Fisher Scientific), and reconstituted in DW at 10 μg solids/μL. The reconstituted beads (20 μL) were then added to the alkylated sample followed by ethanol (EtOH; FUJIFILM Wako Pure Chemical Corp.) to bring the final concentration to 75% (*v*/*v*), with mixing for 20 min. The beads were subsequently immobilized on a magnetic rack. The supernatant was discarded, and the pellet was rinsed with 80% EtOH and 100% acetonitrile (ACN; Thermo Fisher Scientific). The beads were then resuspended in 50 μL of 50 mM Tris-HCl (pH 8.0) (Sigma-Aldrich) with 1 µg trypsin/Lys-C Mix (Promega) and digested by gentle agitation at 37 °C overnight. The digested sample was acidified with 150 μL of 0.1% trifluoroacetic acid (TFA; Thermo Fisher Scientific) and then desalted using GL-Tip SDB (GL Sciences Inc., Tokyo, Japan) according to the manufacturer’s instructions, followed by drying with a centrifugal evaporator. The dried peptides were redissolved in 3% ACN and 0.1% TFA. The redissolved sample was assayed for peptide concentration using a Lunatic instrument (Unchained Labs, Pleasanton, CA, USA) and transferred to a hydrophilic-coated, low-adsorption vial (ProteoSave vial; AMR Inc., Tokyo, Japan).

### 3.4. DIA-MS-Based Proteome Analysis

Peptides (500 ng) from each treatment was directly injected onto a 75 μm × 40 cm PicoFrit emitter (New Objective, Woburn, MA, USA) packed in-house with C18 core-shell particles (CAPCELL CORE MP 2.7 μm, 160 Å material; Osaka Soda Co., Ltd., Osaka, Japan) at 50 °C and then separated with a 120-min gradient at 100 nL/min using an UltiMate 3000 RSLCnano LC system (Thermo Fisher Scientific). Peptides eluted from the column were analyzed on an Orbitrap Exploris 480 Mass Spectrometer (Thermo Fisher Scientific) for overlapping window DIA-MS [14,15]. MS1 spectra were collected in the range of 495–745 *m*/*z* at a 15,000 resolution to set an automatic gain control target of 3 × 106 and maximum injection time of “auto”. MS2 spectra were collected at >200 *m/z* at a 45,000 resolution to set an automatic gain control target of 3 × 106, maximum injection time of “auto”, and stepped normalized collision energy of 22%, 26%, and 30%. The isolation width for MS2 was set to 4 *m*/*z*, and overlapping window patterns of 500–740 *m*/*z* were used for window placements optimized by Skyline (20.2.0.34, University of Washington, Seattle, WA, USA).

The MS files were searched against human and bovine spectral libraries using Scaffold DIA (2.2.1, Proteome Software, Inc., Portland, OR, USA). The human and bovine spectral libraries were generated from the human protein sequence database (UniProt id UP000005640, reviewed, canonical) and the bovine protein sequence database (UniProt id UP000009136, reviewed and unreviewed, canonical) by Prosit (https://www.proteomicsdb.org/prosit/ (accessed on 10 November 2020)) [16,17]. The Scaffold DIA search parameters were as follows: experimental data search enzyme, trypsin; maximum missed cleavage sites, 1; precursor mass tolerance, 10 ppm; fragment mass tolerance, 10 ppm; and static modification, cysteine carbamidomethylation. The protein identification threshold was set at <1% for both peptide and protein false discovery rates. The peptide quantification was calculated by the EncyclopeDIA algorithm [18] in Scaffold DIA. Proteins for which unique peptides were identified in humans and bovine were distinguished from human and bovine proteins, respectively. For each peptide, the four highest-quality fragment ions were selected for quantitation. The quantitative protein value was estimated from the summed peptide quantitative values. Raw data files of the LC-MS/MS analyses have been deposited in the ProteomeXchange Consortium (http://proteomecentral.proteomexchange.org (accessed on 26 February 2021)) via the jPOST partner repository (http://jpostdb.org (accessed on 26 February 2021)) [19] with the dataset identifier PXD24272.

### 3.5. Data Analysis

The secreted proteins were referred to the Human Protein Atlas (https://www.proteinatlas.org/ (accessed on 11 December 2020)). The thresholds for altered proteins were a more than 1.5-fold change and *p* < 0.05 (Welch test) that differed between the two groups. The GO enrichment analysis web tool was used (http://geneontology.org/ (accessed on 17 November 2020)).

## 4. Conclusions

To achieve in-depth proteome analysis from the FBS-containing cell culture supernatant, we investigated the depletion of FBS-derived albumin and the reduction of the medium volume as methods to reduce the dynamic range of protein concentration in the supernatant. As a result, we succeeded in identifying more than 3700 cell-derived proteins by depleting albumin from the supernatant cultured in 2 mL of medium in a 9.5-cm dish. Furthermore, the reproducibility of the entire proteome analysis was improved by the two approaches to reduce the dynamic range. As a demonstration of the power of our proteome analysis, we analyzed the supernatant of TNF-stimulated HeLa cells and non-stimulated HeLa cells. We confirmed that some inflammatory cytokines were elevated in the supernatant of the TNF-stimulated HeLa cells. Based on these results, we believe that our proteome analysis is a powerful method for discovering physiologically active proteins and biomarkers from an FBS-containing cell culture supernatant.

## Data Availability

The data presented in this study are openly available in [ProteomeXchange Consortium (http://proteomecentral.proteomexchange.org)], reference number [PXD24272].

## Supplementary Materials

The following are available online at https://www.mdpi.com/1422-0067/22/5/2565/s1, Table S1: List of human proteins identified before and after (Alb) depletion from the supernatant cultured in 10-mL medium containing 10% FBS, Table S2: List of human proteins lost by albumin depletion, Table S3: List of human proteins observed in albumin-depleted supernatants (sup) of HeLa cells in 10, 5, and 2 mL of medium containing 10% FBS. Table S4: List of human proteins observed in TNF-stimulated and non-stimulated HeLa cells by albumin depletion, Table S5: List of human proteins observed in TNF-stimulated and non-stimulated HeLa cells by albumin non-depletion, Figure S1: Dead and apoptotic rate of HeLa cells cultured in 10, 5, or 2 mL of medium containing 10% FBS for 24 h, Figure S2: Reproducibility of proteome analyses by different treatments, Figure S3: Molecular function GO enrichment analysis of the significantly regulated proteins by TNF stimulation.

## Abbreviations

LC-MS/MS	liquid chromatography–tandem mass spectrometry
FBS	fetal bovine serum
DIA	data-independent acquisition mode
GO	gene ontology
CV	coefficient of variation
TNF	tumor necrosis factor
MEM	minimum essential media
FITC	fluorescein isothiocyanate
PBS	phosphate buffered saline
PI	propidium iodide
Tris	tris (hydroxymethyl) aminomethane
HCl	hydrochloric acid
SDS	sodium dodecyl sulfate
DW	distilled water
EtOH	ethanol
ACN	acetonitrile
TFA	trifluoroacetic acid
