# Supplementary material for: A Simple Method for In-Depth Proteome Analysis of Mammalian Cell Culture Conditioned Media Containing Fetal Bovine Serum
Source: Int J Mol Sci. 2021 Mar 4;22(5):2565. doi: 10.3390/ijms22052565 (PMC7961985; doi:10.3390/ijms22052565)
Supplement: Supplementary file 1 [file ijms-22-02565-s001.zip › Supplementary Figures.pdf]

Supplementary Figures

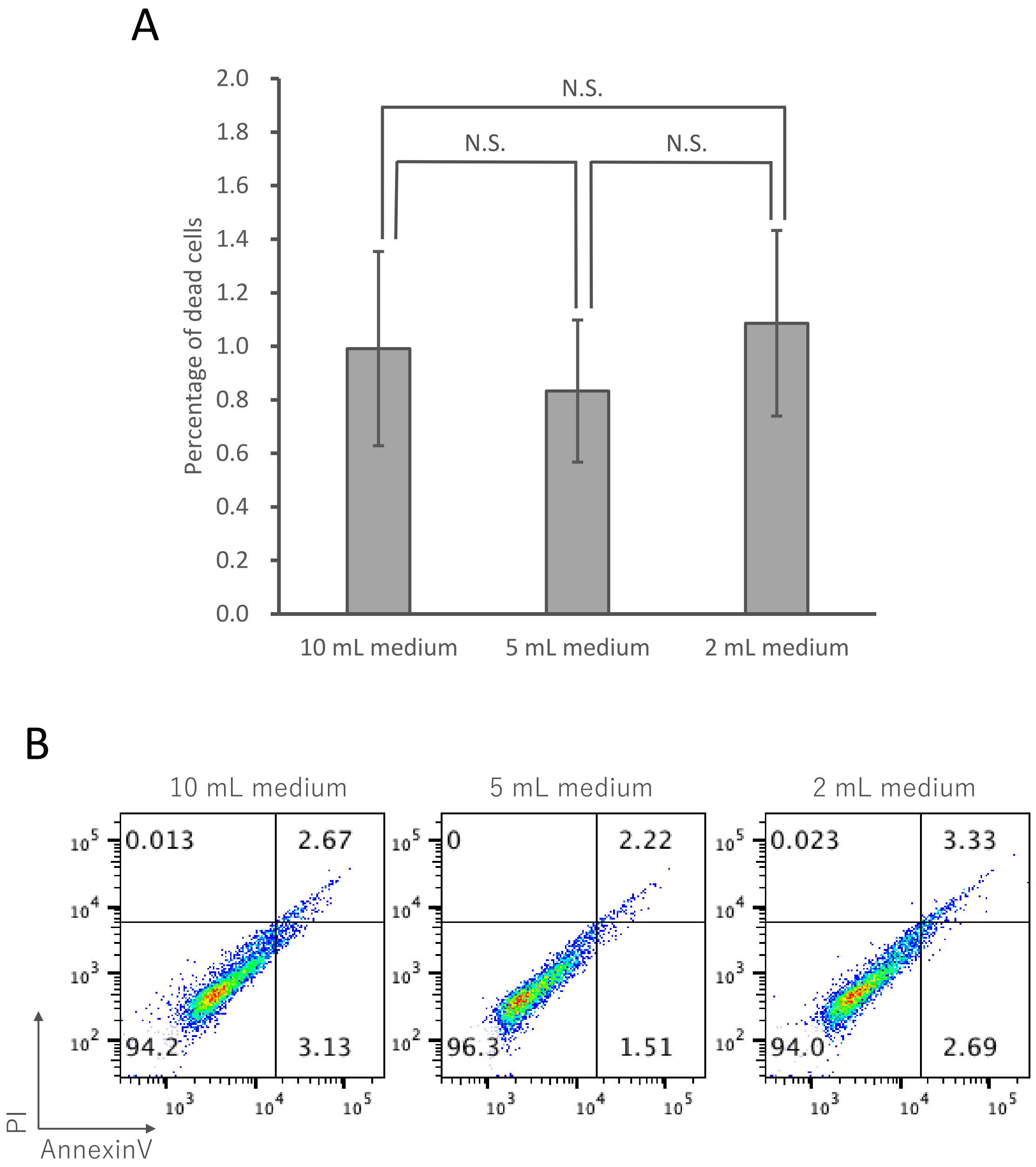

Supplementary Figure 1

Supplementary Figure 1 – Dead and apoptotic rate of HeLa cells cultured in 10, 5, or 2 mL of medium containing 10% FBS for 24 h. (A) The number of dead cells was counted using trypan blue. (B) FACS analysis for FITC-Annexin V-based apoptosis detection. The top right (Annexin V+ / PI+) indicates apoptotic cells. Abbreviations: N.S., not significant; PI, propidium iodide

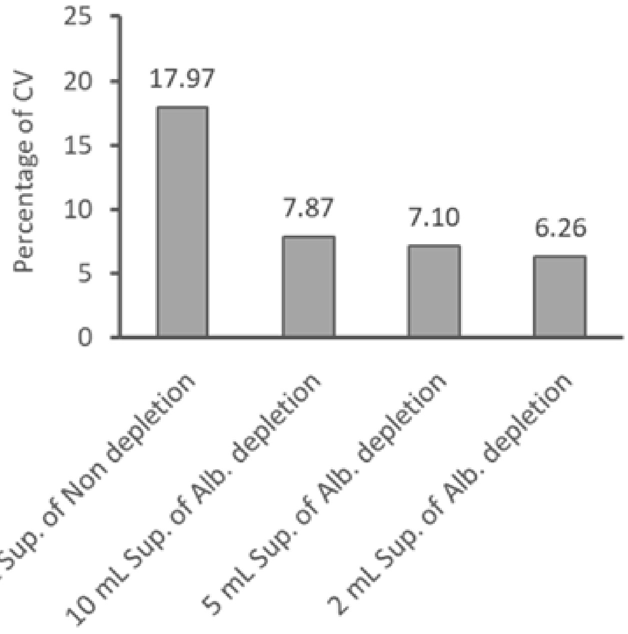

Supplementary Figure 2

Supplementary Figure 2 – Reproducibility of proteome analyses by different treatments. The medians of the protein intensities′ CVs were calculated from the four treatments (n=3 each). The supernatants cultured in 10 mL of the medium were used for non-depletion. The supernatants cultured in 10, 5, and 2 mL of the medium were used for albumin depletion. Abbreviations: ALB, serum albumin; Sup, supernatant

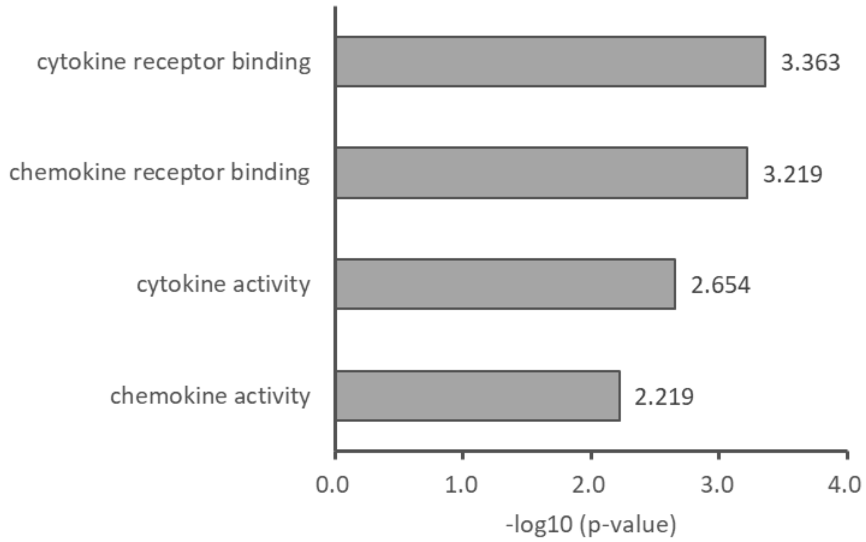

Supplementary Figure 3

Supplementary Figure 3 – Molecular function GO enrichment analysis of the significantly regulated proteins by TNF stimulation. The proteins were found to contain proteins with roles related to cytokines and chemokines.
